# Supplementary material for: Inborn Errors in the LRR Domain of Nod2 and Their Potential Consequences on the Function of the Receptor
Source: Cells. 2021 Aug 9;10(8):2031. doi: 10.3390/cells10082031 (PMC8392326; doi:10.3390/cells10082031)
Supplement: Supplementary file 1 [file cells-10-02031-s001.zip › cells-1312479-supplementary/cells-1312479-supplementary resubmitted/Supplementary figure S1.pdf]

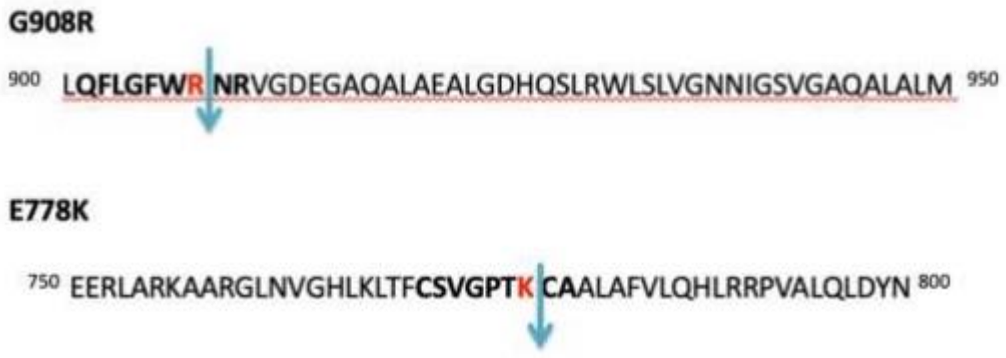

Supplementary Figure S1: The two new residues in position 908 and 778 of NOD2, introduce new peptide cleavage sites.
