# Supplementary material for: Inborn Errors in the LRR Domain of Nod2 and Their Potential Consequences on the Function of the Receptor
Source: Cells. 2021 Aug 9;10(8):2031. doi: 10.3390/cells10082031 (PMC8392326; doi:10.3390/cells10082031)
Supplement: Supplementary file 1 [file cells-10-02031-s001.zip › cells-1312479-supplementary/cells-1312479-supplementary resubmitted/supplementary Figure S3-revised -dynamut.pdf]

Supplementary Figure S3. The changes in the vibrational entropy and interatomic interactions upon mutations in the LRR domain of nod2. (A) Amino acids colored according to the vibrational entropy change upon mutation. BLUE represents a rigidification of the structure and RED a gain in flexibility. Images are provided y Dynamut. (B) Interatomic interactions. Residues are presented by light-green sticks among their surrounding residues involving in different interactions. Images are provided y Dynamut.

A

R702W

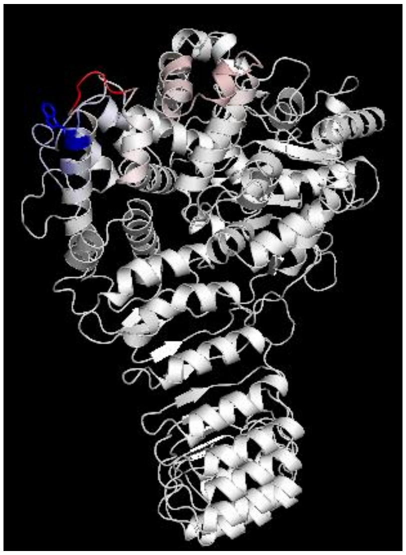

B

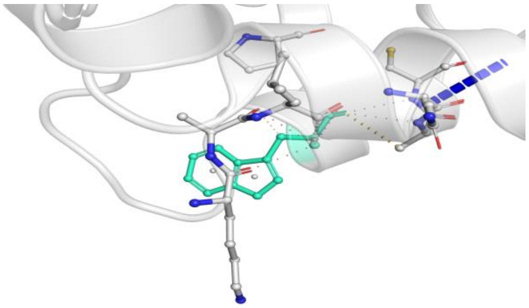

Interatomic reactions of the mutant residue

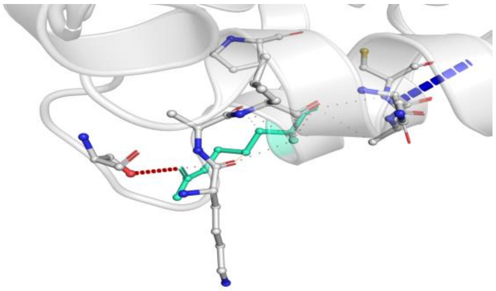

Interatomic reactions of the wild residue

A725G

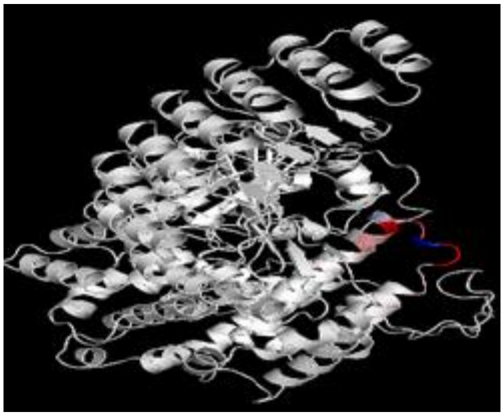

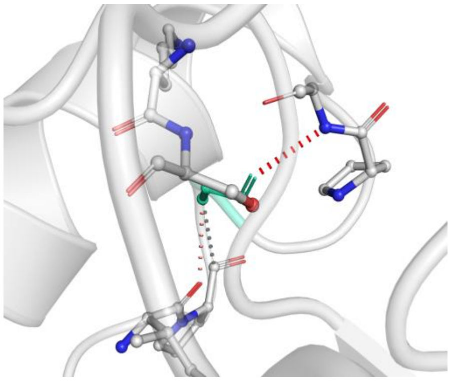

Interatomic reactions of the mutant residue

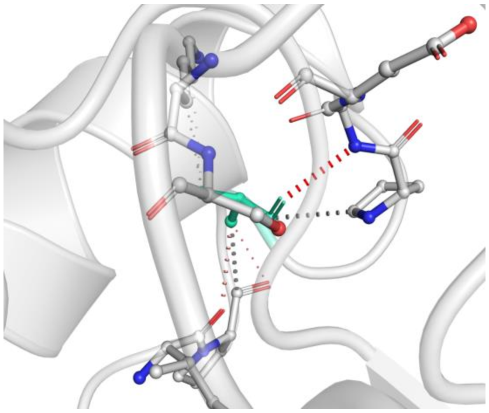

Interatomic reactions of the wild residue

A755V

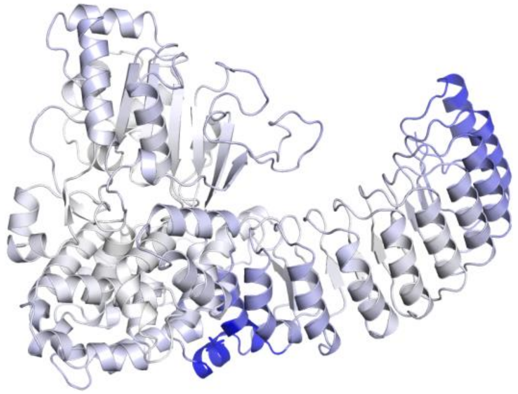

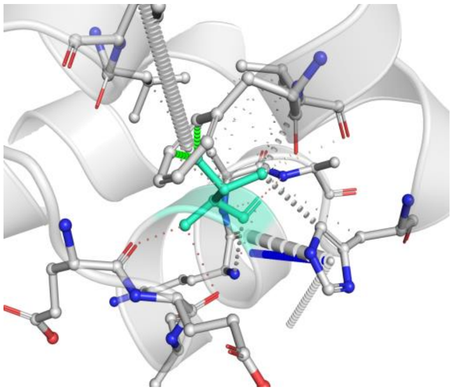

Interatomic reactions of the mutant residue

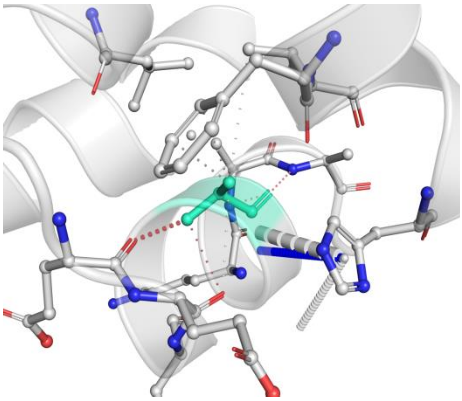

Interatomic reactions of the wild residue

A885T

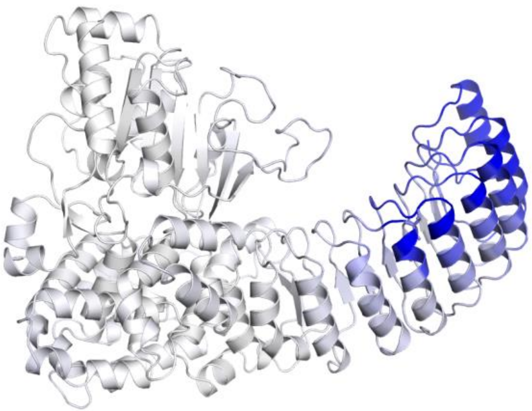

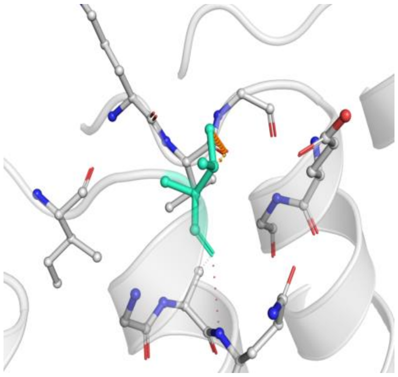

Interatomic reactions of the mutant residue

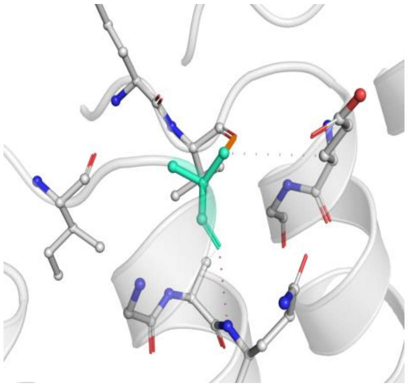

Interatomic reactions of the wild residue

G843K

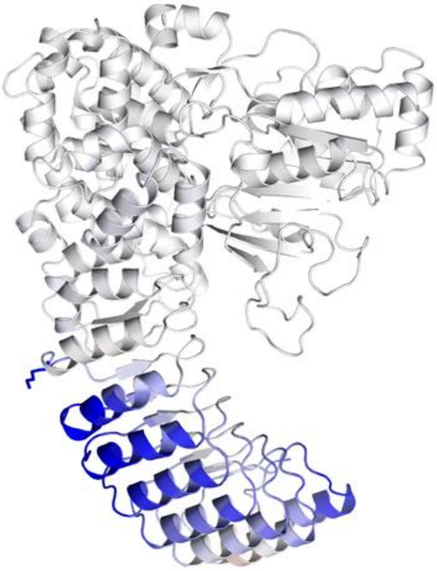

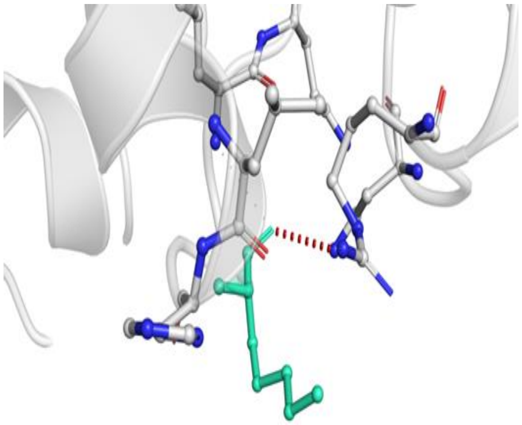

Interatomic reactions of the mutant residue

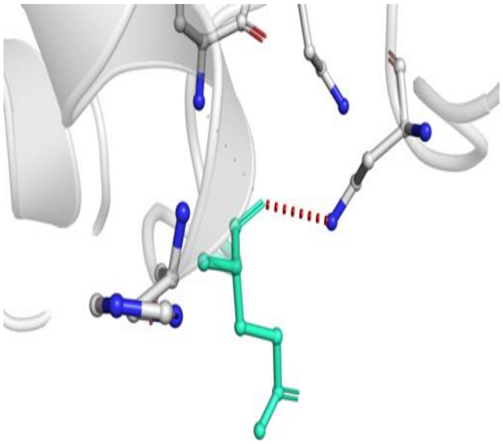

Interatomic reactions of the wild residue

G924D

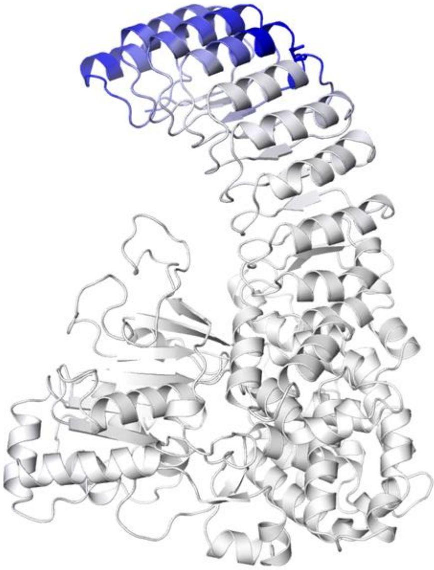

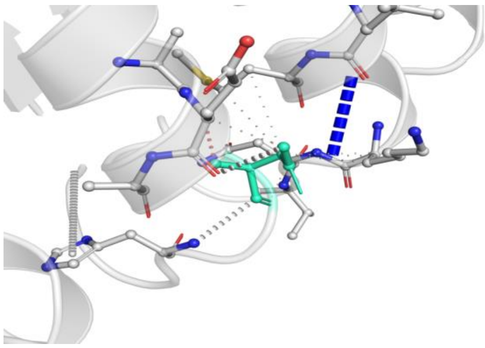

Interatomic reactions of the mutant residue

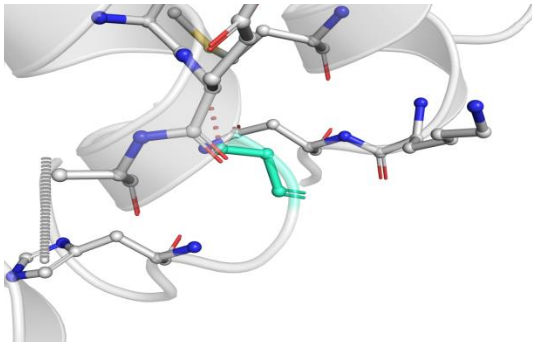

Interatomic reactions of the wild residue

M863V

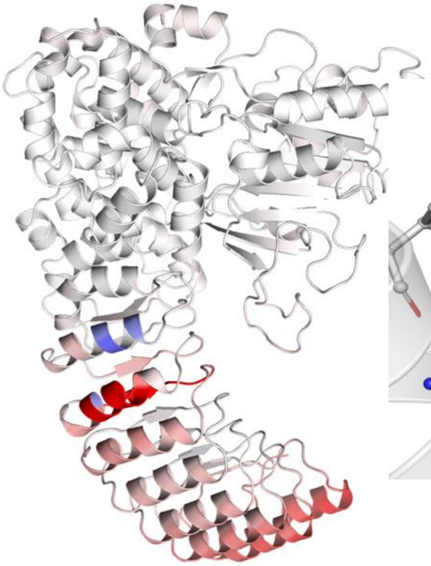

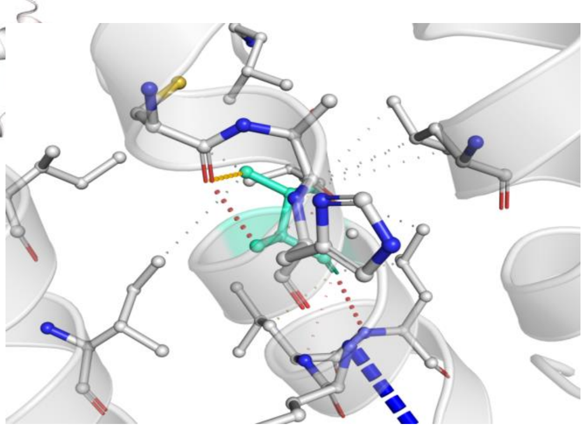

Interatomic reactions of the mutant residue

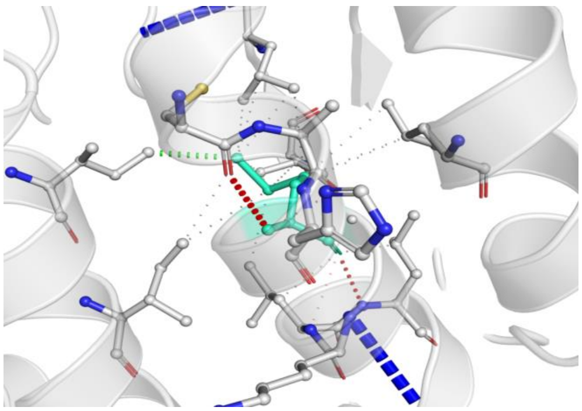

Interatomic reactions of the wild residue

N852S

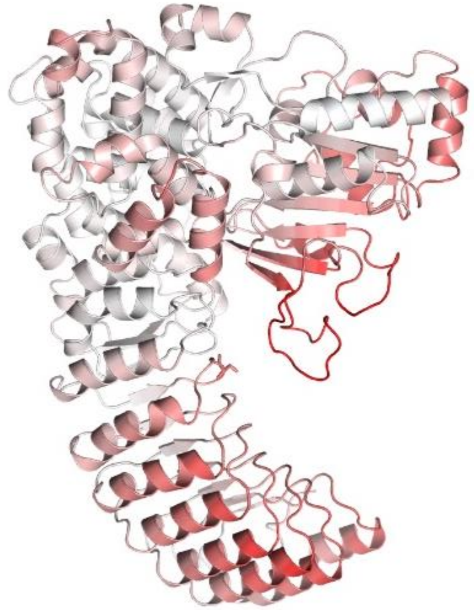

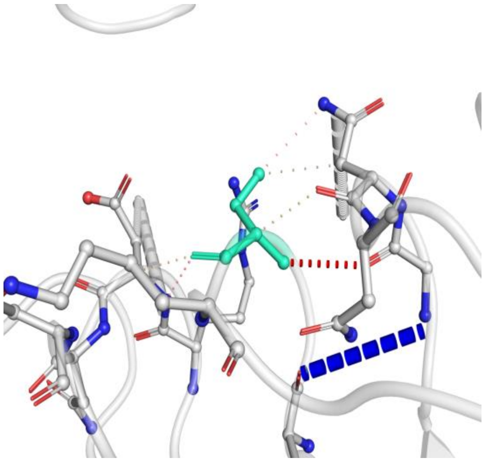

Interatomic reactions of the mutant residue

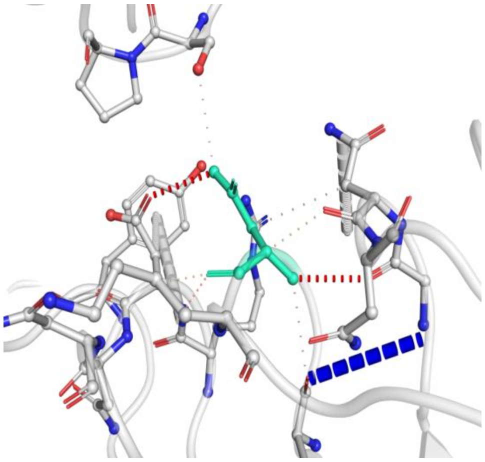

Interatomic reactions of the wild residue

P268S

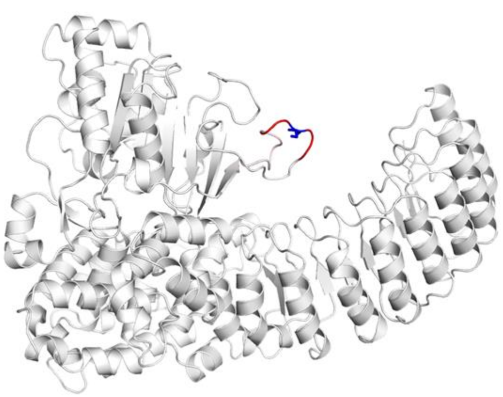

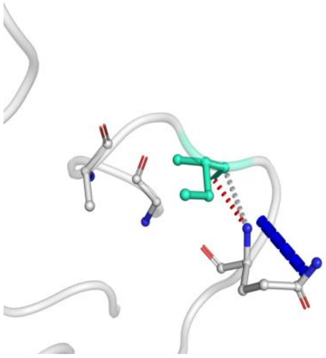

Interatomic reactions of the mutant residue

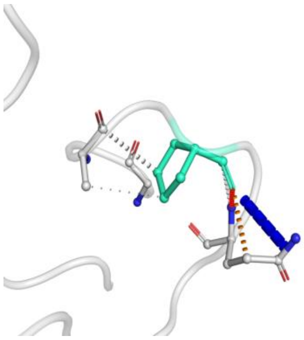

Interatomic reactions of the wild residue

R760C

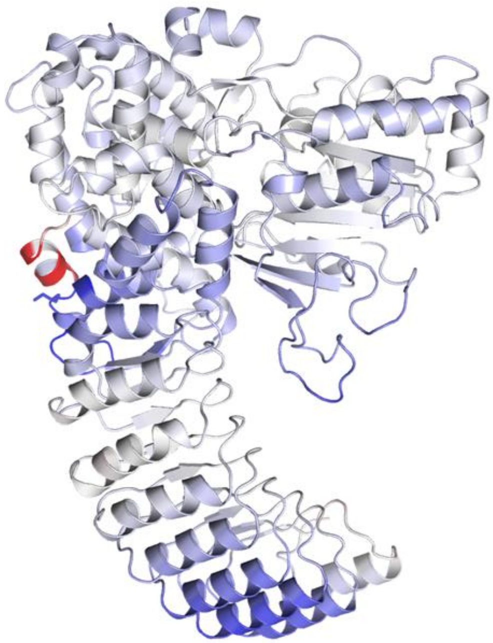

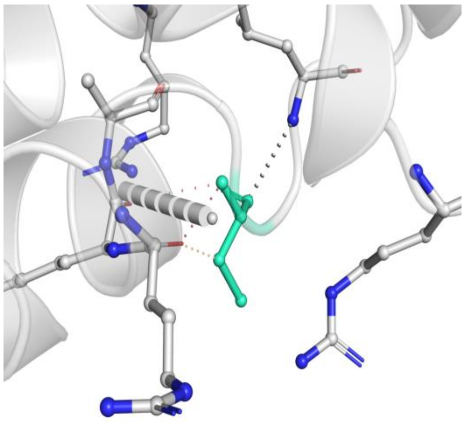

Interatomic reactions of the mutant residue

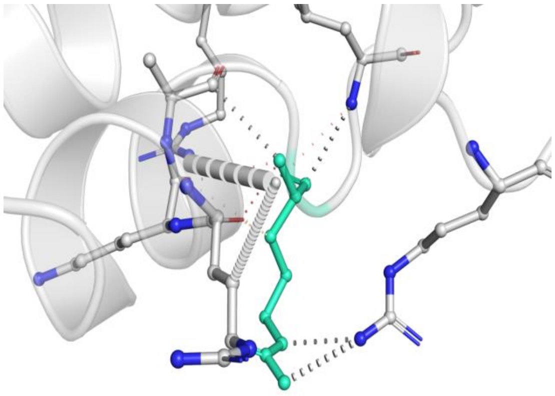

Interatomic reactions of the wild residue

R790w)

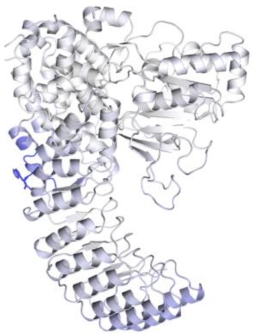

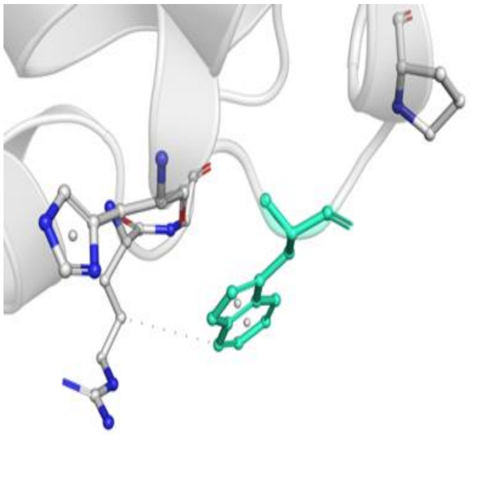

Interatomic reactions of the mutant

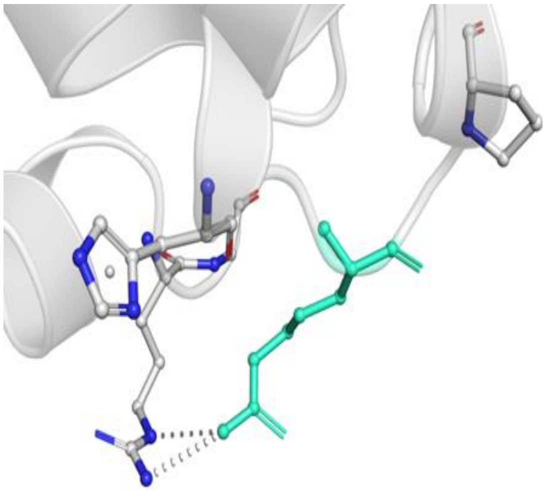

Interatomic reactions of the wild residue

V793M

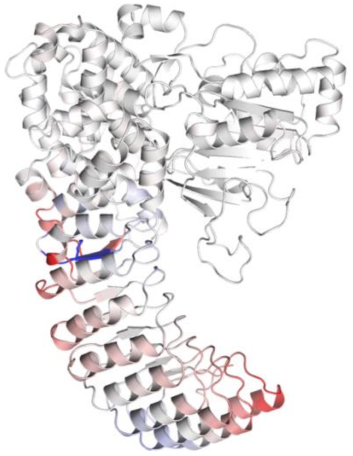

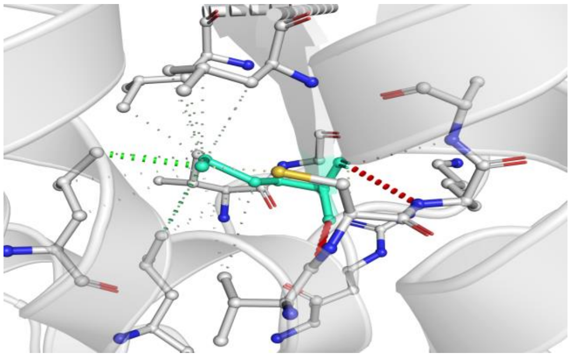

Interatomic reactions of the mutant residue

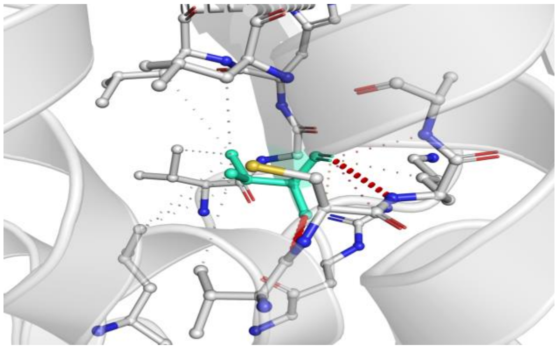

Interatomic reactions of the wild residue

E778k

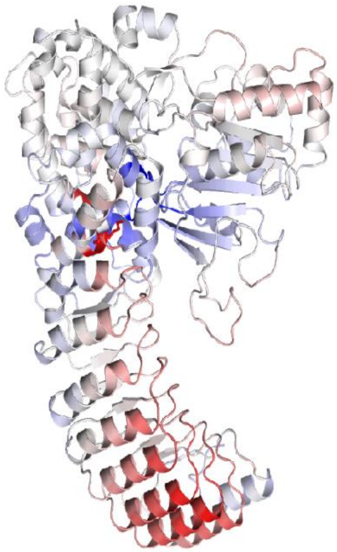

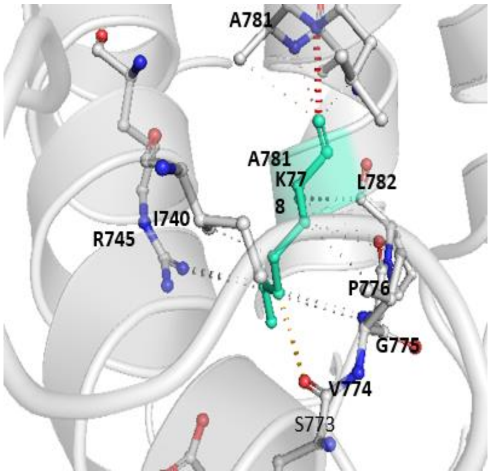

Interatomic reactions of the mutant residue

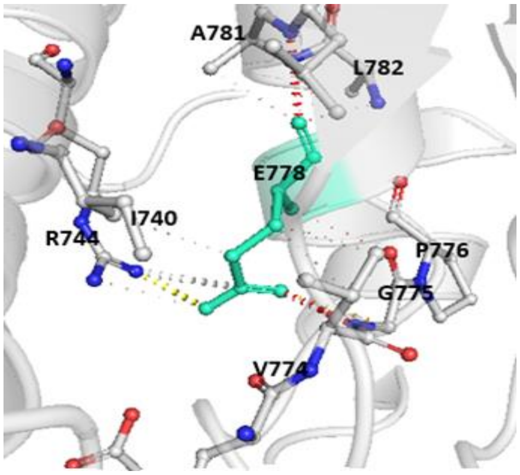

Interatomic reactions of the wild residue

G908R

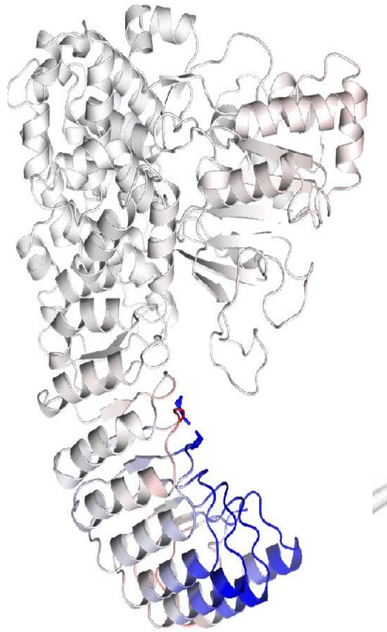

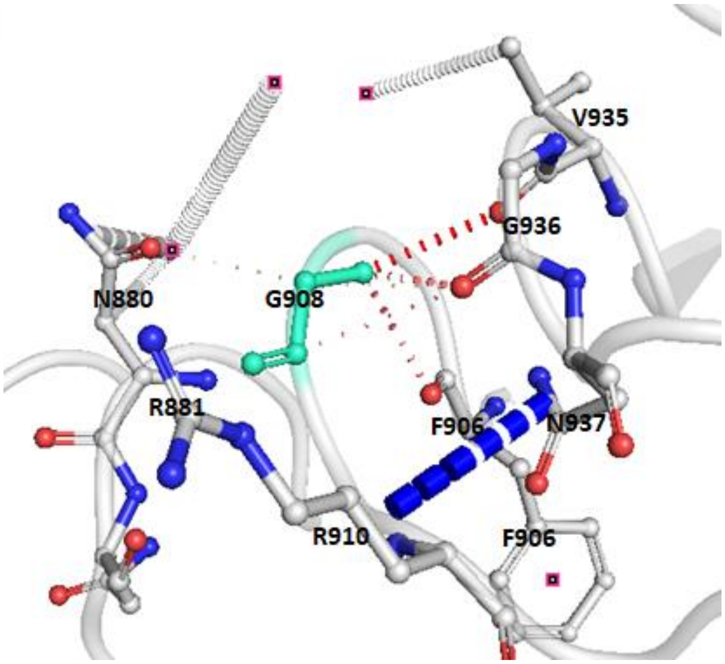

Interatomic reactions of the mutant residue

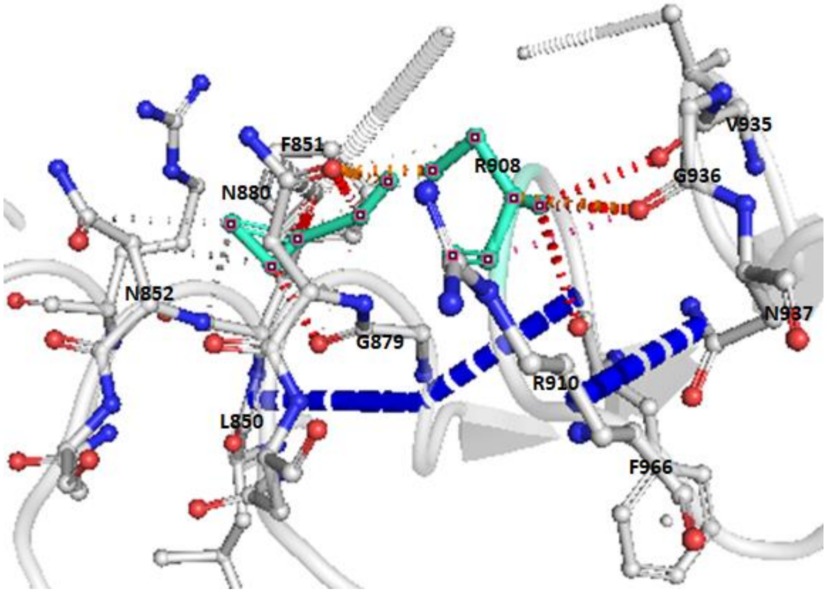

Interatomic reactions of the wild residue

Color definition for contacts ▲

Bond Type	Color
Hydrogen bonds	
Water mediated hydrogen bonds	
Weak hydrogen bonds	
Water mediated weak hydrogen bonds	
Halogen bonds	
Ionic interactions	
Metal complex interactions	
Aromatic contacts	
Hydrophobic contacts	
Carbonyl contacts	
