# Supplementary material for: Inborn Errors in the LRR Domain of Nod2 and Their Potential Consequences on the Function of the Receptor
Source: Cells. 2021 Aug 9;10(8):2031. doi: 10.3390/cells10082031 (PMC8392326; doi:10.3390/cells10082031)
Supplement: Supplementary file 1 [file cells-10-02031-s001.zip › cells-1312479-supplementary/cells-1312479-supplementary resubmitted/supplementary file-revised-1.pdf]

1- Material and Methods:

1-1- Data mining

We explored db SNP NCBI [1] and Expasy [2] for the SNPs located in the LRR domain of NOD2 and selected 14 SNPs that have been reported in association with diseases. These SNPs were used for the further analysis.

The FASTA format of the NOD2 sequence was extracted from Uniprot17. The secondary structures and the solvent accessibility of the residues in the wild and mutant proteins were extracted from the PDP files by DSSP18 and Getarea19, 20 respectively.

1-2- Characterization of the potential damaging effects of the SNPs

The potential damaging effects of the mutations were evaluated by the Provean (Protein Variation Effect Analyzer) analysis tool (http://provean.jcvi.org/index.php) [3].

Provean predicts tolerated and damaged SNPs based on the multiple alignment information for every position of the sequence [3].

In this work, the FASTA SEQ obtained from NCBI and the mutated positions were submitted as a query to Provean. The substitutions at each position equal to or less than a threshold of −2.28 indicate an intolerant or damaged variant.

1-3- Prediction of the functional consequence of the SNPs

To determine the potential effects of these SNPs on the function of the NOD2 receptor, we used the PolyPhen-2 (Polymorphism Phenotyping) computational tool (http://coot.embl.de/PolyPhen) [4]. For this analysis, the amino acid sequence of NOD2 wild type in fasta format was used as input query and the position and substitution of each of the two SNPs were submitted to PolyPhen.

PolyPhen estimates the effects of amino acid substitutions using a set of basic algorithms that are built based on the sequence and structural and phylogenetic information of the protein and calculates a Position-Specific Independent Counts (PSIC) profile score. PSIC is a fast technique for calculation of position-specific sequence weights in a multiple sequence alignment (MSA) [5]. Finally, PolyPhen appraised the mutations qualitatively based on a cutoff PSIC score: probably damaging (PSIC score > 0.85), possibly damaging (PSIC score > 0.15), or benign.

For further assessment of the functional effects of SNPs, we used the PANTHER (http://www.pantherdb.org) tool [6]. PANTHER works based on HMM statistical models and evolutionary relationships. This tool uses the information from the sequence conservations within a hidden Markov model (HMMs) to predict the functional effects of the missense variants [6].

The amino acid substitution and protein fasta sequence were used as inputs to PANTHER, and a score was computed by the tool, based on the position-

specific evolutionary preservation (PSEP) score. Based on the selected thresholds, SNPs were characterized: “probably damaging" (time > 450my), "possibly damaging" (450my > time > 200my), and "probably benign" (time < 200my).

We also assess the effects of SNPs regarding their location in the functional or structural conserved regions with Conserf webtool [7]. This computational tool works based on the phylogenetic relationships between homologous sequences and measures the evolutionary conservation of the SNPs in a protein sequence [7].

1-4- Determination of the SNPs’ structural effects on protein stability and flexibility

To analyze the consequence of the point mutations on the structural stability of the protein, I-Mutant [8] was used. I-Mutant is a computational tool that predicts the stability of a mutant protein using a ProTerm-derived dataset. We submitted the SNPs of the NOD2 sequence in fasta format as input. We also performed further analysis using DynaMut [9] to assess the flexibility and interatomic interactions in wild and mutant proteins.

For a deeper analysis of the structural effects of the correspondence SNPs, we used HOPE web service [54]. HOPE is a web-server tool that analyzes the consequence of SNPs on the structure and conformation of a protein. The NOD2 wild type and the SNPs sequences were individually used as the input to the HOPE server.

1-5- Potential post-translational modification (PTM) sites

The potential PTM sites were searched in the human NOD2 protein using NetPhos [61] which predicts the Serine, Threonine, and Tyrosine phosphorylation sites in a given protein sequence. For searching the ubiquitination sites and peptide cleavage sites, we used BDM-PUB [10] and ProP 1.0 [11] respectively.

1-6- Network analysis

For further analysis of the effects of these SNPs, we analyzed the interaction of the mutant NOD2 with other downstream proteins.

Protein–protein interaction (PPI) networks were constructed and analyzed by STRING and Cytoscape v. 3.4.0 [12] . Briefly, NOD2 was introduced to the software as the seed protein, and PPI networks were constructed using STRING [13] and visualized and analyzed in Cytoscape. STRING extracts information from both the experimental and predicted interactions based on gene neighborhood, gene co-occurrence, gene fusions, gene co-expression, and literature mining and extracts highly interconnected regions (clusters) and their related pathways [12].

References

1. Sherry, S.T.; Ward, M.-H.; Kholodov, M.; Baker, J.; Phan, L.; Smigielski, E.M.; Sirotkin, K. dbSNP: the NCBI database of genetic variation. *Nucleic Acids Res.* **2001**, *29*, 308–311.

2. Gasteiger, E.; Gattiker, A.; Hoogland, C.; Ivanyi, I.; Appel, R.D.; Bairoch, A. ExPASy: the proteomics server for in-depth protein knowledge and analysis. *Nucleic Acids Res.* **2003**, *31*, 3784–3788.

3. Choi, Y.; Chan, A.P. PROVEAN web server: a tool to predict the functional effect of amino acid substitutions and indels. *Bioinformatics* **2015**, *31*, 2745–2747.

4. Adzhubei, I.; Jordan, D.M.; Sunyaev, S.R. Predicting functional effect of human missense mutations using PolyPhen-2. *Curr. Protoc. Hum. Genet.* 2013, *76*, 7.20. 21–27.20. 41.

5. Sunyaev, S.R.; Eisenhaber, F.; Rodchenkov, I.V.; Eisenhaber, B.; Tumanyan, V.G.; Kuznetsov, E.N. PSIC: profile extraction from sequence alignments with position-specific counts of independent observations. *Protein Eng.* **1999**, *12*, 387–394.

6. Mi, H.; Muruganujan, A.; Ebert, D.; Huang, X.; Thomas, P.D. PANTHER version 14: more genomes, a new PANTHER GO-slim and improvements in enrichment analysis tools. *Nucleic Acids Res.* **2019**, *47*, D419–D426.

7. Ashkenazy, H.; Abadi, S.; Martz, E.; Chay, O.; Mayrose, I.; Pupko, T.; Ben-Tal, N. ConSurf 2016: an improved methodology to estimate and visualize evolutionary conservation in macromolecules. *Nucleic Acids Res.* **2016**, *44*, W344–W350.

8. Capriotti, E.; Fariselli, P.; Casadio, R. I-Mutant2. 0: predicting stability changes upon mutation from the protein sequence or structure. *Nucleic Acids Res.* **2005**, *33*, W306–W310.

9. Rodrigues, C.H.; Pires, D.E.; Ascher, D.B. DynaMut: predicting the impact of mutations on protein conformation, flexibility and stability. *Nucleic Acids Res.* **2018**, *46*, W350–W355.

10. Cai, B.; Jiang, X. Computational methods for ubiquitination site prediction using physicochemical properties of protein sequences. *BMC Bioinform.* **2016**, *17*, 116.

11. Duckert, P.; Brunak, S.r.; Blom, N. Prediction of proprotein convertase cleavage sites. *Protein Eng. Des. Sel.* **2004**, *17*, 107–112.

12. Shannon, P.; Markiel, A.; Ozier, O.; Baliga, N.S.; Wang, J.T.; Ramage, D.; Amin, N.; Schwikowski, B.; Ideker, T. Cytoscape: a software environment for integrated models of biomolecular interaction networks. *Genome Res.* **2003**, *13*, 2498–2504.

13. Szklarczyk, D.; Gable, A.L.; Lyon, D.; Junge, A.; Wyder, S.; Huerta-Cepas, J.; Simonovic, M.; Doncheva, N.T.; Morris, J.H.; Bork, P. STRING v11: protein–protein association networks with increased coverage, supporting functional

discovery in genome-wide experimental datasets. *Nucleic Acids Res.* **2019**, *47*, D607–D613.
