# Supplementary material for: Inborn Errors in the LRR Domain of Nod2 and Their Potential Consequences on the Function of the Receptor
Source: Cells. 2021 Aug 9;10(8):2031. doi: 10.3390/cells10082031 (PMC8392326; doi:10.3390/cells10082031)
Supplement: Supplementary file 1 [file cells-10-02031-s001.zip › cells-1312479-supplementary/cells-1312479-supplementary resubmitted/supplementary proofback-Figure S2.pdf]

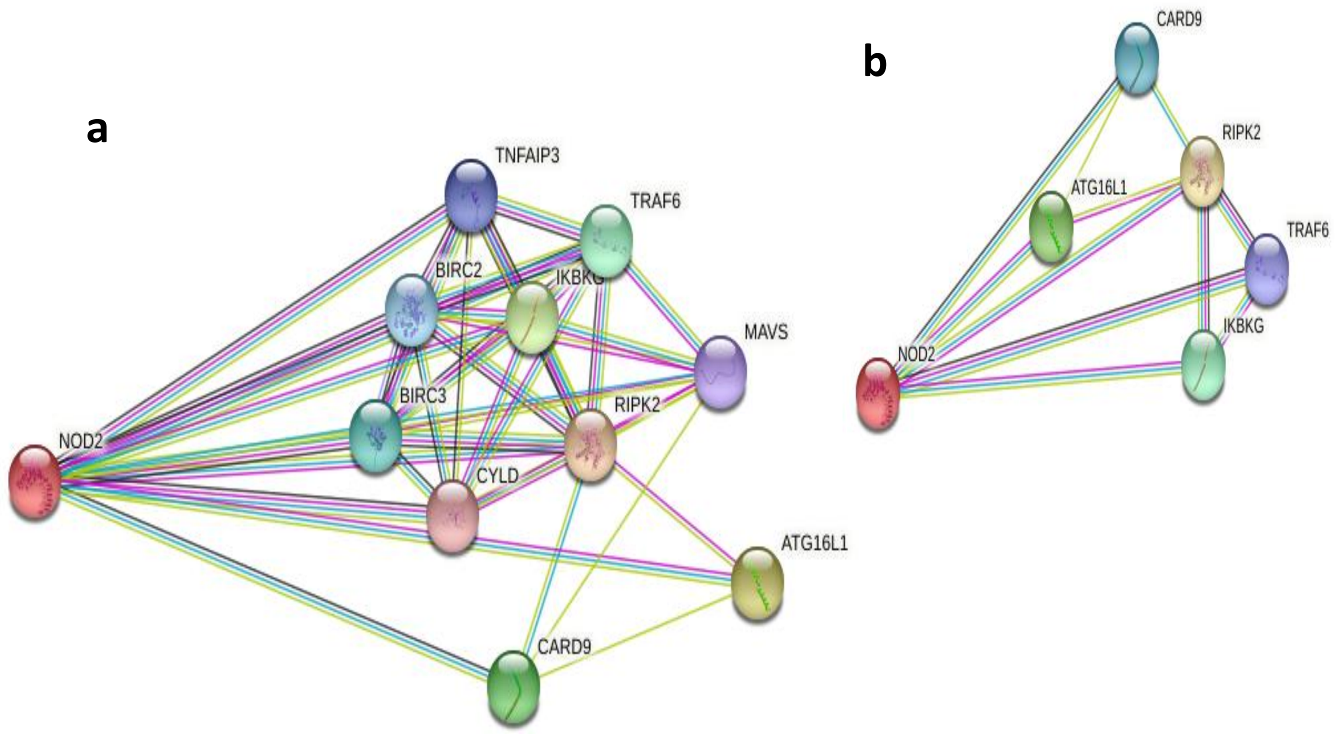

c

KEGG Pathways	Description
hsa05131	Apoptosis
hsa04621	NOD-like receptor signaling pathway
hsa04622	RIG-I-like receptor signaling pathway
hsa05152	Tuberculosis
hsa05222	Small cell lung cancer

Figure S2. (a) The PPI network of the NOD2 receptor. (b) PPI networks were constructed, and the cluster contained the highly connected regions in the network identified by the Molecular COmplex DEtection plugin. (c) The related pathways of these highly connected proteins were identified and extracted. Nodes represent proteins and edges represent their interactions.
