# Supplementary material for: Inborn Errors in the LRR Domain of Nod2 and Their Potential Consequences on the Function of the Receptor
Source: Cells. 2021 Aug 9;10(8):2031. doi: 10.3390/cells10082031 (PMC8392326; doi:10.3390/cells10082031)
Supplement: Supplementary file 1 [file cells-10-02031-s001.zip › cells-1312479-supplementary/cells-1312479-supplementary resubmitted/supplementary table S1-hope.pdf]

Supplementary Table S2. Prediction of the effects of SNPs on the structure and interatomic interactions in the mutant receptors by HOPE Project.

Residues	**	*	Conservation	Properties
R702W	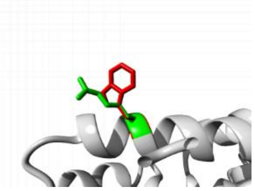	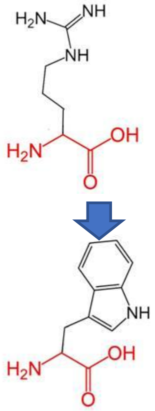	Damaging	-The mutant residue is bigger; this might lead to bumps.<br>-The wild-type residue charge was POSITIVE; the mutant residue charge is NEUTRAL.<br>-The mutant residue is more hydrophobic than the wild-type residue. This can result in loss of hydrogen bonds and/or disturb correct folding.
A725G		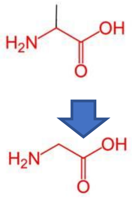	Damaging	-The mutant residue is smaller; this might lead to loss of interactions.<br>- Glycine is very flexible and can disturb the required rigidity of the protein at this position.<br>- The wild residue is more hydrophobic. Hydrophobic interactions, either in the core of the protein or on the surface, will be lost.
A755V	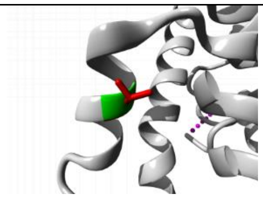	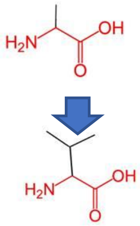	Damaging	-The wild and mutant residue differs in charge, and hydrophobicity.<br>-The mutant residue is bigger and is<br>-less compatible with an α-helices as secondary structure in this region.
R760C	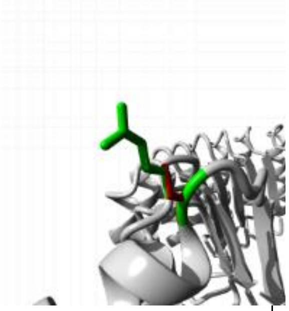	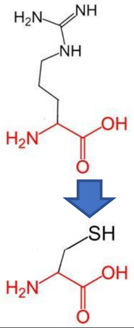	Not damaging	-The mutant residue is smaller than the wild-type residue this might lead to loss of interactions<br>-The wild-type residue charge was positive; the mutant residue charge is NEUTRAL.<br>-The mutant residue is more hydrophobic than the wild-type residue which result in loss of hydrogen bonds and/or disturb correct folding.
E778K	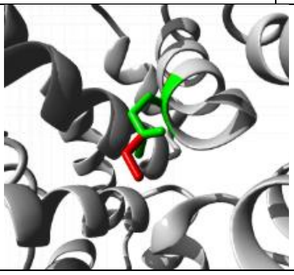	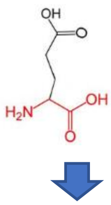	damaging	-The mutant residue is bigger which might lead to bumps.<br>-The wild-type residue charge was NEGATIVE; the mutant residue charge is POSITIVE. This opposite charge may cause repulsion with other residues in the protein or ligands.

		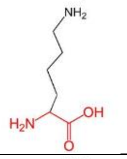		
R790W	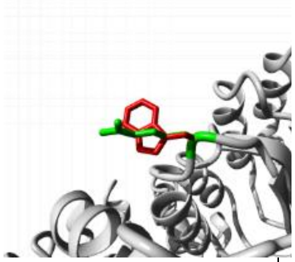	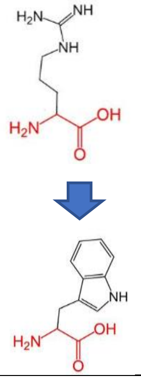	Not damaging	-The mutant residue is bigger than the wild-type residue this might lead to bumps.<br>-The wild-type residue charge was positive; the mutant residue charge is NEUTRAL.<br>-The mutant residue is more hydrophobic than the wild-type residue This can result in loss of hydrogen bonds and/or disturb correct folding.
V793M	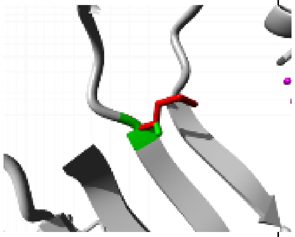	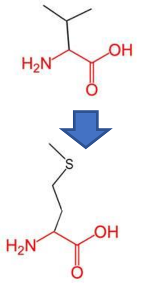	Not damaging	The mutant residue is bigger, this might lead to bumps
E843K	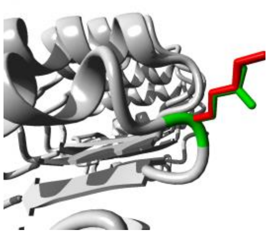	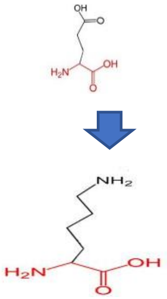	Not damaging	-The mutant residue is bigger than the wild-type residue.<br>-The wild-type residue charge was NEGATIVE, the mutant residue charge is POSITIVE. . This can cause repulsion with other residues in the protein or ligands.
N852S	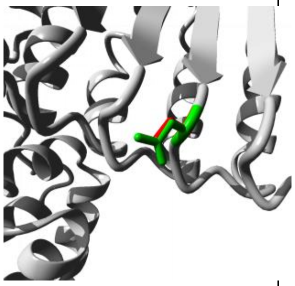	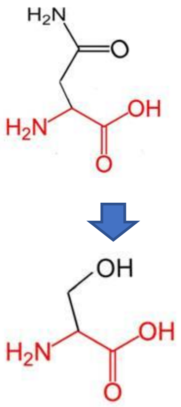	Damaging	- The mutant residue is smaller; this might lead to loss of interactions.<br><br>-The mutant residue is more hydrophobic than the wild type. This can result in loss of hydrogen bonds and/or disturb correct folding.

M863V	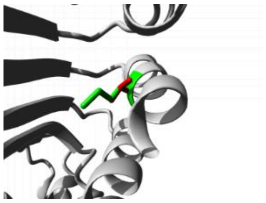	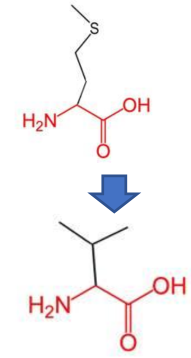	Not damaging	-The mutant residue is smaller; this might lead to loss of interactions.
A885T	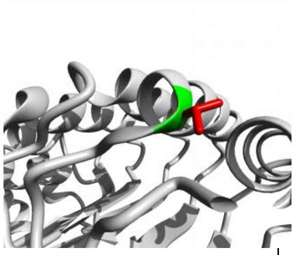	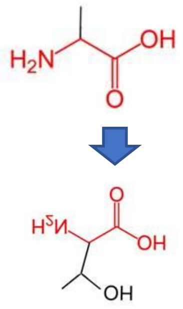	Not damaging	-The mutant residue is bigger than the wild-type residue.<br>-The wild-type residue is more hydrophobic than the mutant residue.<br>-The mutant residue is less compatible with an α-helices as secondary structure in this region.
G908R	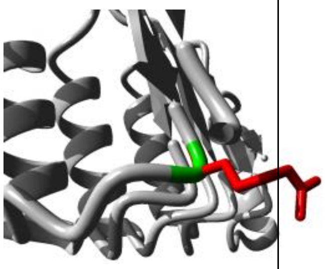	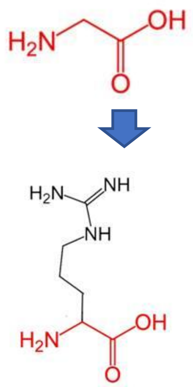	Damaging	- The mutant residue is bigger; this might lead to bumps.<br>- mutation introduces a charge, this can cause repulsion of ligands or other residues with the same charge<br>-The torsion angles for this residue are unusual. only glycine is flexible enough to make these torsion angles, mutation into another residue will force the local backbone into an incorrect conformation and will disturb the local structure<br>- The wild-type residue is a glycine, the most flexible of all residues. This flexibility might be necessary for the protein's function. Mutation of this glycine can abolish this function.
G924D	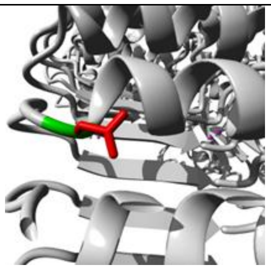	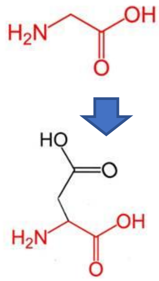	Damaging	-The wild-type residue charge was neutral; the mutant residue charge is NEGATIVE. This can cause repulsion of ligands or other residues with the same charge.<br>-The wild-type residue is more hydrophobic than the mutant residue.<br>-The mutant residue is bigger; this might lead to bumps.<br>-The torsion angles for this residue are unusual. only glycine is flexible enough to make these torsion angles, mutation into another residue will

				force the local backbone into an incorrect conformation and will disturb the local structure

* original (up) and the mutant (down) amino acid. The backbone, which is the same for each amino acid, is colored red. The side chain, unique for each amino acid, is colored black. ** The protein is colored grey, the side chains of both the wild-type and the mutant residue are shown and colored green and red respectively.
