# Supplementary material for: Inborn Errors in the LRR Domain of Nod2 and Their Potential Consequences on the Function of the Receptor
Source: Cells. 2021 Aug 9;10(8):2031. doi: 10.3390/cells10082031 (PMC8392326; doi:10.3390/cells10082031)
Supplement: Supplementary file 1 [file cells-10-02031-s001.zip › cells-1312479-supplementary/cells-1312479-supplementary resubmitted/supplementary table S2-revised.pdf]

Supplementary Table S1. Prediction of SNPs effects on the stability of mutated protein obtained by I-MUTANT 2.0 DDG < 0: Decrease Stability; DDG > 0: Increase Stability [89].

**LRR Domain**	**I-mutant**		
**SNPs**	**Stability (SVM2)**	**RI**	**DDG- (kcal/mol)**
A725G	Decreased	7	−1.23
A755V	Decreased	3	−0.23
R760C	Decreased	6	−0.79
E778K	Decreased	7	−0.96
R790W	Decreased	6	−0.2
V793M	Decreased	7	−1.27
E843K	Decreased	6	−0.36
N852S	Decreased	4	−0.11
M863V	Decreased	7	−0.77
A885T	Stabilize	8	0.76
G908R	Decreased	6	−1.23
G924D	Decreased	4	−0.78
P268S	Decreased	8	−1.48

RI: Reliability Index: 0 < RI < 10. Calculations with relatively high **reliability indices** will be expected to perform well.
